# The Draft Genome Sequence of *Paenibacillus polymyxa* Strain CCI-25 Encompasses High Potential for Secondary Metabolite Production

**DOI:** 10.1128/genomeA.00366-16

**Published:** 2016-05-19

**Authors:** Gajender Aleti, Livio Antonielli, Erika Corretto, Branislav Nikolić, Angela Sessitsch, Günter Brader

**Affiliations:** Bioresources Unit, Health and Environment Department, Austrian Institute of Technology (AIT), Tulln, Austria

## Abstract

We report here the draft genome sequence of *Paenibacillus polymyxa* strain CCI-25, which displays strong antifungal and antibacterial activities *in vitro*. The genome encompasses nonribosomal peptide synthetases predicted to encode a tridecaptin, polymyxin, fusaricidin, an iturin-like synthetase, a lantibiotic similar to paenicidin A, as well as a type 1 polyketide synthase.

## GENOME ANNOUNCEMENT

Plant-associated *Paenibacillus polymyxa* strains are well noted for their production of a wide range of secondary metabolites (1–3), predominantly lipopeptides and polyketides involved in plant growth promotion and biocontrol of fungi (4–6). Here, we highlight the secondary metabolite capacity of *P. polymyxa* strain CCI-25 isolated from vermicompost. Both colonies and lipopeptide and polyketide crude extracts (7, 8) exhibited strong antimicrobial activity against *Escherichia coli* and fungi, including *Fusarium oxysporum* ACC01, *Botrytis cinerea* ofi 501-E (Austrian Institute of Technology [AIT] collection), and *Rhizoctonia solani* CBS101769, on plate assays.

To evaluate the molecular basis for secondary metabolite production, genomic DNA was isolated by phenol-chloroform extraction, and a library was prepared, according to the manufacturer’s protocol, using the Nextera XT kit (Illumina, San Diego, CA). Library sequencing was performed using an Illumina MiSeq platform (MiSeq reagent kit version 3). Sequencing generated 2,213,773 paired-end reads with 124 ± 53-fold coverage after PhiX sequence removal by Bowtie2 (9). Adapter and quality trimming were performed using Trimmomatic-0.32 (10). Overlapping reads were merged with FLASH (11), and paired-end reads were assembled by SPAdes 3.1.0 (12). Quality control of mapping data was carried out by Qualimap 2.2 (13), and assembly quality was estimated by QUAST 3.2 (14). Assembly resulted in 117 contigs >1,000 bp, with an *N*_50_ size of 95,765 bp. The draft genome size is 5.61 Mb, with a G+C content of 44.95%. The identification of 40 highly conserved single-copy marker genes in the assembly by PhyloSift version 1.0.1 (15) indicated completeness of the genome and excluded contaminant sequences. Genomic BLAST showed similarities to *P. polymyxa* CR1. The NCBI Prokaryotic Genome Annotation Pipeline (PGAP) identified 5,146 genes, 4,953 coding sequences (CDSs), 15 complete 5S rRNAs, 30 partial 16S rRNAs, 37 partial 23S rRNAs (for a total of 15 putative rRNA operons), 107 tRNAs, 4 noncoding RNAs (ncRNAs), and 241 pseudogenes. The rRNAs were further confirmed by RNAmmer 1.2 (16). Prediction of secondary metabolite-encoding sequences was performed by antiSMASH (17).

The CCI-25 draft genome encompasses nonribosomal peptide synthetases with sequence similarities to published genes (1, 18–22), and the prediction includes the encoding of a tridecaptin with valine instead of isoleucine at the 13th position compared to *Paenibacillus terrae* NRRL B-30644 and fusaricidin C, and a polymyxin with leucine instead of phenylalanine at the 6th position compared to *P. polymyxa* M1. In addition, an iturin- and paenilarvin-like compound with altered monomer composition (d-Gly-d-Orn-d-Glu-d-nrp-l-nrp-l-Ile-l-Val) compared to the published metabolites from *Bacillus amyloliquefaciens* FZB42 (<58% identity) and *Paenibacillus larvae* DSM 25430 (<40% identity) (1, 23) has been predicted. CCI-25 contains a lantibiotic gene similar to paenicidin A and a predicted polyketide synthase with a different number of acyl carrier domains with 61% identity to bacillaene synthase from *B. amyloliquefaciens* FZB42 and 87% identity to *P. polymyxa* M-1 polyketide synthase (23). Given the fact that about 370 kb (6.6% of the total genome) is dedicated to secondary metabolite biosynthesis, CCI-25 has high potential to be exploited for medical or agricultural applications.

### Nucleotide sequence accession number. 

The nucleotide sequences have been deposited at the DDBJ/EMBL/GenBank under the accession no. LTYJ00000000. The version described in this paper is the first version.
